# Risk factors for completed suicide among people who use drugs: A scoping review protocol

**DOI:** 10.12688/hrbopenres.13098.3

**Published:** 2021-05-17

**Authors:** Lisa Murphy, Suzi Lyons, Michael O'Sullivan, Ena Lynn

**Affiliations:** 1 Health Research Board, Dublin 2, Ireland

**Keywords:** Suicide, people who use drugs, substance-related disorders, risk factors, scoping review

## Abstract

*Background:* Research over the past several decades has shown an increased risk for completed suicide among people who use drugs (PWUD). However, no study to date has attempted to summarise the available literature on the variety of risk factors associated with this increased risk. This paper presents a protocol for a scoping review that aims to systematically map and synthesise the extent and nature of published, unpublished and grey literature related to risk factors for suicide among PWUD.

*Methods:* The following six-stage methodological framework for scoping reviews proposed by Arksey and O’Malley with enhancements by Levac and colleagues will be used: (1) identifying the research question, (2) identifying relevant studies, (3) study selection, (4) charting/mapping the data, (5) collating, summarising and reporting results and (6) expert consultation. The review will be conducted and reported in accordance with the PRISMA Extension for Scoping Reviews (PRISMA-ScR). Key inclusion and exclusion criteria will be developed to guide literature screening and data charting. Three reviewers will conduct the initial screening of published, unpublished and grey literature. Identified risk factors will be collated, summarised and categorised iteratively by two independent reviewers. Stakeholder consultation will occur with experts from a national steering committee, a national advisory group, a national suicide prevention centre and a European drug monitoring centre.

*Conclusion:* Collating and thematically categorising the various risk factors for suicide among this high-risk group will hold important implications for future research, policy and practice. The research will be disseminated through publication in a peer-reviewed academic journal and a conference presentation, and by sharing the findings with key stakeholders working within research, policy-making and professional practice contexts.

## Introduction

Suicide is a significant public health concern. According to the World Health Organisation (WHO), over 800,000 people die by suicide every year, which amounts to approximately one suicide death every 40 seconds ^1, 2^ and 34 million years of life lost annually ^3^. In 2016, suicide accounted for 1.4% of all deaths worldwide, making it the 18th leading cause of death globally ^4^.

In addition to the significant emotional toll experienced by the families, friends and communities of people who die by suicide ^5, 6^, there are considerable economic costs to suicide incurred by individuals, families and society more generally. This includes direct monetary costs linked to suicide (e.g. the cost of emergency services, medical care, medicolegal costs and funeral expenses) and indirect costs associated with loss of life (e.g. loss in productive activity and loss of earnings due to premature mortality) ^7– 9^. In its Comprehensive Mental Health Action Plan ^10^, the WHO commit to reducing global suicide mortality by 10% between 2012 and 2020. Under objective three of this plan (‘ *to implement strategies for the promotion and prevention in mental health*’), a defined action for WHO Member States is the development and implementation of a comprehensive national suicide prevention strategy, with particular emphasis on vulnerable groups ^3^.

Among those populations known to be at increased risk of suicide are people who use drugs (PWUD) ^11– 13^. Evidence from epidemiological and clinical research indicates a 7- to 22-fold increase in suicide mortality among PWUD relative to that expected in the general population ^14– 17^. The factors that contribute to suicide are complex, wide-ranging and multi-faceted ^18, 19^, acting at multiple levels (i.e. individual, familial, communal and societal) and varying across groups and over time ^20^. Here, we outline a protocol for a scoping review with the primary aim of providing a comprehensive overview of existing literature on risk factors for completed suicide, specifically among PWUD. The particular objectives of the review are:

(a) To map the extent, range and nature of available evidence on risk factors for suicide among PWUD.
(b) To identify knowledge gaps and limitations in this body of literature and make recommendations for addressing them.
(c) To inform suicide prevention policy and best practice guidelines for working with PWUD.

### Study rationale

A scoping review approach was deemed suitable for several reasons. Notwithstanding several (unsystematic) literature reviews ^13, 18, 21– 24^ on risk factors for suicide among PWUD, and previous systematic reviews ^12, 17, 25– 27^ and meta-analyses ^2, 15, 28^ that aimed to quantify the association of problem drug use with suicide mortality, no study has sought to systematically identify and thematically map the available evidence on risk factors for suicide among PWUD. This is surprising, given the breadth of empirical research on the predictors, patterns, outcomes and implications of problem drug use ^29– 33^, academic consensus that problem drug use remains a significant risk factor for suicide ^14, 16^ and widespread recognition of PWUD as a high-risk group for suicide in national and international reports and suicide prevention strategies ^34, 35^.

Consequently, there is limited clarity on the extent (i.e. size and breadth), range (i.e. variety) and nature (i.e. characteristics and contexts) ^36^ of the evidence regarding risk factors for suicide among PWUD, as well as ambiguity regarding the overall progress and direction of this field of research. Scoping reviews are an increasingly popular form of knowledge synthesis that aim to systematically search and map the breadth of available evidence (including evidence in published and grey literature), categorise key concepts, identify knowledge gaps and research deficits, and propose recommendations to guide future research ^37, 38^. In this sense, a scoping review is an ideal approach toward a comprehensive understanding of suicide among PWUD, particularly given the breadth of grey literature (e.g. policy papers, governmental and organisational reports, etc.) on this topic. Moreover, limited clarity on risk factors for suicide among PWUD has implications for policy and practice; understanding risk and the contexts in which risk may be amplified are critical precursors to developing targeted interventions and prevention strategies for any group, including PWUD ^39^. A key characteristic of the scoping review is the incorporation of stakeholder consultation into the methodological framework to both inform and validate the study findings ^39^. This process provides opportunity for knowledge transfer and exchange with experts working at the intersection of research, policy and practice.

## Protocol

The review will be guided by the methodological framework for scoping reviews outlined by Arksey and O’Malley ^40^, with subsequent enhancements by Levac and colleagues ^39^. This framework involves six stages, which are discussed in further detail below: (1) identifying the research question, (2) identifying relevant studies, (3) selecting studies, (4) mapping/charting the data, (5) collating, summarizing, and reporting the results, and (6) expert consultation. The scoping review will also be reported in accordance with the Preferred Reporting Items for Systematic Reviews and Meta-Analyses (PRISMA) Extension for Scoping Reviews (PRISMA-ScR) ^36^.

### Stage 1: Identifying the research question

Scoping review research questions are expected to be broad enough in nature to capture the breadth of research on a given topic ^40^, while also encompassing a clearly articulated scope of enquiry ^39^. As per PRISMA-ScR guidelines ^36^, the research question was guided by the Population, Concept, Context (PCC) mnemonic ^41^, albeit tailored to include the outcome of interest for the purposes of this scoping review. Thus, the following research question was identified based on the overarching aim of the scoping review: What is the extent, range, and nature of evidence regarding risk factors for suicide among PWUD? Addressing this research question will allow us to ascertain current knowledge gaps and research deficits in this field of research and propose recommendations for future research, policy and practice.

***Population.*** The review team note the variety of terms used to describe PWUD in this body of literature. For the purposes of the scoping review, PWUD is considered an umbrella phrase under which various terms indicative of problem drug use are subsumed, including, but not limited to, any of the following:

(a) People who use, misuse, or abuse drugs (including non-medical use of prescribable drugs and illicit drug use).
(b) People with a diagnosis of substance use disorder (SUD) / drug use disorder (DUD).
(c) People with drug dependence.
(d) People who are regular or ‘casual’ users of drugs.
(e) People who report recent drug use.

***Concept.*** For the purposes of the scoping review, a risk factor is defined as any characteristic, fixed or modifiable, that makes it more likely that an individual will die by suicide ^38^. Given the wide range of possible risk factors ^34^, the scope of the review will be kept deliberately broad so that risk factor categories emerge during the process of the systematic search and data charting. However, risk factors for suicide can be broadly grouped as occurring at the individual, relationship, community, society, and systemic levels ^34^. We anticipate that some evidence sources may not explicitly identify a correlate or predictor of suicide as a risk factor per se, and so decisions on the parameters of what constitutes a risk factor will be made iteratively as we become increasingly familiar with the breadth and nature of the field.

***Outcome.*** In relation to suicide as an outcome for PWUD, studies may refer to one or more of the following:

(a) Suicide, defined as “Different manners of non-natural death have different numbers of undetermined cases in terms of intent; for example, in a hanging or a shooting it is usually easy to differentiate between a suicide or a trauma (or a crime), while for drowning, traffic accidents or intoxication it is more difficult. Circumstantial findings, such as suicide notes, expressed intent or other findings such as self-inflicted cutting of the wrist followed by drowning, are suggestive of the intent” (E950-953, X60-84-ICD–International Classification of Disease–9 and 10).
(b) Undetermined suicide, defined as “When crime can be ruled out and it cannot be established whether the manner of death is a suicide or an accident, the manner of death is recorded as death of undetermined intent” (E980, Y10-34, ICD–International Classification of Disease–9 and 10).
(c) Probable suicide. Following previous recommendations ^42– 44^, all deaths with a diagnosis of suicide or undetermined suicide can be considered probable suicide deaths.

The scoping review methodology is an iterative process ^39^, and so the research question may be refined, or additional questions identified, as the review team become increasingly familiar with the body of literature.

### Stage 2: Identifying relevant research

***Information sources and search strategy.*** A comprehensive search strategy to identify relevant literature will be developed in consultation with a health information specialist, and in accordance with the Joanna Briggs Institute (JBI) Reviewer’s Manual for scoping reviews ^41^. The strategy will involve systematic searching of published, unpublished and grey literature, and the entire search strategy, including the rationale behind any decisions made, will be included in the final manuscript.

As recommended ^41^, a three-step strategy will be utilised to identify published literature. To ensure that all appropriate index terms (i.e. MeSH), keywords and phrases are included in the main search, Step 1 involves an initial limited search of two electronic databases and an analysis of the keywords and phrases contained in the titles and abstracts of retrieved papers, as well as of the index terms used to describe the articles. The following two electronic databases will be searched: Medline (EBSCO) and CINAHL (EBSCO). Search strings combining keywords, phrases and index terms using Boolean operators will be developed in collaboration with a health information specialist. In Step 2, the search strings will be adapted and applied across all included databases, which are: Medline (EBSCO), CINAHL (EBSCO), PsycINFO (Ovid), SOCIndex (EBSCO), the Cochrane Database of Systematic Review and the Campbell Collaboration Database of Systematic Reviews. Several key academic journals will be identified by the review team and hand searched for relevant published articles that may not be returned in database searching. This step includes initial screen of all titles/abstracts returned from database searching, followed by full-text screening of all evidence sources that meet the eligibility criteria (see below). Following full-text screening, Step 3 involves searching the reference lists of all evidence sources included in the review for additional sources missed in Step 2.

The review team are aware of the breadth of potential sources of grey and unpublished literature. To ensure a systematic search of grey and unpublished literature relating to risk factors for suicide among PWUD, a number of steps will be taken. First, grey literature databases (e.g. Open Grey) will be searched using keywords and phrases identified in published literature. As per previous recommendations ^45, 46^, only the first 100 hits (as sorted by relevance) from searches performed using grey literature search engines will be screened, as further screening will unlikely result in additional relevant literature. Second, specific types of evidence sources will be sought and screened, including abstracts submitted to flagship conferences on both substance abuse and suicide, postgraduate theses and dissertations (e.g. Electronic Theses Online Service), preprints (e.g. OSF Preprints), policy documents and governmental and organisational reports. Finally, the review team will attempt to contact academic experts, professional societies and relevant organisations to ascertain the availability of any additional evidence sources not identified in previous searches of published, unpublished or grey literature.

***Eligibility criteria.*** All peer-reviewed and non-peer-reviewed articles and reports published in the English language will be eligible for inclusion in the review. Careful consideration was given to the timeframe of the search; as one objective of the scoping review is to make recommendations for policy and practice, more up-to-date research was considered most appropriate. Therefore, searches will be limited to evidence sources published over the past 20 years, between 2000 and 2020, inclusive. However, particularly pertinent studies that remain relevant to today’s literature (i.e. seminal papers published before the year 2000) will be identified in the full text searching and data extraction stages and considered for inclusion in the review on a case by case basis according to the search and inclusion criteria. Review articles that provide new insights will be eligible for inclusion. This includes evidence syntheses, narrative reviews, rapid reviews, systematic reviews and meta-analyses. No limitations will be placed on study design (i.e. cross-sectional, case-control, cohort, prospective, retrospective studies etc. will all be eligible for inclusion). Where the review team identify evidence sources with obvious overlap in either participant samples (e.g. multiple publications from the same prospective study) or datasets (e.g. studies or reports that draw from the same dataset or information system), sources that provide the most information relevant to the aims of the scoping review will be prioritised. Thus, any articles, reports and evidence syntheses that do not provide new information will be excluded.

As per PRISMA-ScR guidelines ^36^, the search strategy will also be underpinned by key eligibility criteria based on the PCC mnemonic ^41^, which again includes the primary outcome of interest for purposes of this scoping review. Inclusion and exclusion criteria relating to (a) Population (PWUD), (b) Concept (risk factors), (c) Outcome (suicide), and (d) Context (region, drug treatment settings, etc.) are listed in Table 1. It is noted that these criteria may be refined throughout the process of the scoping review owing to increasing familiarity with the body of literature and subject matter ^39^.

**Table 1.** Inclusion and exclusion criteria for study selection.

Included	Excluded
*Population: People who use drugs (PWUD)*	
• Sources in which it is made explicit that the participant group (or a subgroup) were PWUD	• Sources in which is it not made explicit that the deceased (or a subgroup) were PWUD
• Sources that include a participant group (or subgroup) who use, abuse or are dependent on drugs *only* OR • Sources that include a participant group (or subgroup) who use, abuse or are dependent on *both* drugs and alcohol	• Sources that include a participant group (or subgroup) who use, abuse or are dependent on alcohol *only* OR • Sources in which the participant group (or a subgroup) is only identified as having substance use problems, which could be related to alcohol alone, drug(s) alone or a combination of both
• Sources involving adult participants or participants in late adolescence	• Sources involving children or early adolescents (below 15) *only*
*Concept: Risk factors*	
• Sources that explicitly identify a variable, or several variables, as risk factors for suicide among PWUD OR • Sources in which risk factors can be inferred (e.g. sources that report sex segregated data)	• Sources that do not explicitly analyse risk factors for suicide among PWUD OR • Sources in which risk factors cannot be inferred
*Outcome: Suicide*	
• Sources in which the primary outcome variable (or one of several outcome variables) is completed suicide	• Sources that focus on suicide ideation, non-fatal attempted suicide, non-fatal deliberate self-harm, or accidental overdose *only* OR • Sources that focus on the cause of mortality among PWUD *only* OR • Sources that focus on the means of suicide death *only*
• Sources that include death via overdose (or poisoning) as an outcome and analyse deliberate overdose (or poisoning) deaths as a distinct subgroup	• Sources in which overdose (or poisoning) is a primary outcome but intentionality is not made explicit (i.e. no differentiation between intentional or accidental overdose deaths)
*Context*	
• Sources that provide insight into risk factors for suicide among PWUD across all settings, including before, during and after drug treatment, psychiatric treatment and incarceration, and other legal or social care contexts	• Sources in which the illicit use of a drug or drugs was solely to complete suicide (i.e. intentional injecting of insulin, which was not prescribed to the individual, for the purpose of completing suicide)
• Sources from any geographic region	

### Stage 3: Selecting studies

All search results will be imported into Endnote X7 (Mendelay reference manager software can be used as a freely available alternative) and any duplicates removed. As per scoping review guidelines ^39^, reviewers will meet at the start, during and at the end of each stage of selecting studies and any disagreements on article inclusion will be discussed. Firstly, the entire review team will independently apply the eligibility criteria to a random sample of 25 titles/abstracts and then meet to discuss discrepancies and make modifications to the criteria to ensure complete agreement, if required ^41^. Next, one reviewer (LM) will apply the eligibility criteria to all titles/abstracts of all retrieved sources; those that are deemed unsuitable for progression to full-text review will be excluded and the reason for exclusion recorded. Two second reviewers (SL and MO’S) will then independently review all excluded titles/abstracts to ensure accuracy. Results will be compared between all reviewers until consensus is reached. If conflict remains, a fourth reviewer (EL) will be consulted until consensus is reached. Finally, two independent reviewers (LM and SL *or* MO’S) will independently apply the eligibility criteria to full text publications; those that are deemed unsuitable for progression to Stage 4 (mapping/charting the data) will be excluded and the reason for exclusion recorded. Any disagreements will be discussed and, if required, a third reviewer (EL) will be consulted until full consensus on inclusion and exclusion is achieved.

Throughout this selection process, queries for discussion will be recorded and all queries and associated verdicts will be included in the final manuscript as an appendix. Reasons for exclusion of sources following title/abstract and full text review will be reported in the PRISMA-ScR flow diagram in the final manuscript ^38^.

### Stage 4: Mapping/charting the data

In scoping review methodology, data charting is the process of extracting relevant data from sources deemed eligible for inclusion ^39, 40^. A bespoke data charting tool will be developed by the review team a priori, guided by recommendations pertaining to data charting in the JBI Reviewer’s Manual for scoping reviews ^41^ and by the specific aims and objectives of the review. The following types of information will be collected: study characteristics (e.g. year of publication and country), the overall aim/purpose of the study/report, the study design, study/report setting, population characteristics (e.g. age, gender and ethnicity), the use of diagnostic inclusion criteria for drug use (e.g. DSM or other criteria) or the authors definition of drug use, the presence/absence of a control or comparison group, the definition of suicide (e.g. probable or undetermined), the risk factors examined (e.g. correlates or predictors of suicide) and how they were measured, the main findings and information pertaining to the analyses (e.g. adjustments for covariates), the interpretation of the findings, recommendations for future research, policy or practice, and study limitations.

Two independent reviewers (LM and MO’S) will pilot the data chart on a random selection of 10 publications ^39^, and then meet to discuss the comprehensiveness of the data chart and determine accuracy and consistency in the data being extracted. It is expected that data charting will be an iterative process ^39, 40, 47, 48^; although the data chart will be developed a priori, it may be refined throughout the piloting and charting process. Consultation will take place between the reviews throughout the data charting process. Any disagreements will be discussed and, if required, resolved in consultation with a third reviewer (SL or EL). Data charting will be conducted using Microsoft Excel. Authors of studies will be contacted if further clarification of the information in any evidence source is required. The data chart headings will be presented in a summary table in the final manuscript, and all charted data will be made publicly available.

### Stage 5: Collating, summarizing, and reporting the results

The data will be collated and summarised in accordance with the overall aim and objectives of the scoping review and the PRISMA-ScR checklist will be used for reporting the results ^36^. The data will be analysed and aggregated using quantitative (i.e. frequency analysis) and qualitative (i.e. narrative synthesis) methods. First, the characteristics of included studies will be summarised and presented. This includes information pertaining to the geographic distribution, publication dates, types of population samples and methodologies of included evidence sources, as well as the types of evidence sources available (e.g. empirical research, policy documents, reports etc.). Variation in relation to methodology and study design, among other characteristics, is expected. Second, a narrative synthesis of risk factors for suicide among PWUD will be conducted and presented, with a focus on the types of risk factors being examined and the key variables/concepts within each risk factor category. Where possible, trends will be analysed according to specific drugs, as well as the number of drugs reported, albeit this is contingent upon the scope of the individual studies included in the review. We anticipate that certain risk factors (e.g. age range) may vary as a function of other factors (e.g. sex), and this will be a consideration during the data mapping and collating processes. Risk factors will be characterised as modifiable (e.g. current drug use) or non-modifiable (family history of drug use) during data mapping and collation, and distinctions will be drawn between proximal (e.g. polydrug use) and distal (early traumatic experience) risk factors. Moreover, evidence sources will be categorised and analysed according to the description of the population provided. For instance, evidence sources that specifically focus on people with drug dependence or drug use disorder will be analysed separately and compared to those that refer to ‘drug users’ or ‘people who use drugs’, without an indication of the severity of use.

Finally, the results will conclude with a narrative overview of research limitations, knowledge gaps and areas have been under-researched to inform directions for future research and considerations for policy and practice. Recommendations for policy and practice extracted from reports and policy documents will also be charted, summarised and integrated into the review findings. It is anticipated that the process of mapping and analysing the data will be an iterative one, with enrichment and refinement of the review findings and resulting recommendations following expert consultation (see below). The methods used to collate and summarise the data, as well as the rationale behind all decisions pertaining to data handling and analysis, will be described in detail in the final manuscript.

### Stage 6: Expert consultation

Expert consultation is an important component of the scoping review methodology as a means by which to engage important stakeholders with expertise in research, policy and practice, and thus enhance the methodological rigour and applicability of the review ^39^. Consultations will occur with national experts from the Irish National Drug-Related Deaths Index (NDRDI) Steering Committee, which includes community representation for families affected by drug-related deaths, and the Technical Advisory Group of the National Office of Suicide Prevention, as well as international experts from the European Monitoring Centre for Drugs and Drug Addiction (EMCDDA) and the World Health Organization. Finally, experts from other relevant organisations, professional societies, research centres and institutes will be contacted (e.g. the National Suicide Research Foundation in Ireland, the National Drug Research Institute in Australia, and Harm Reduction International). As recommended, the preliminary findings from Stage 5 will be used as a foundation from which to inform consultations ^39^. The purposes of expert consultation in this instance are three-fold. Specifically:

(a) To obtain additional input regarding important evidence sources not identified in published and grey literature searches.
(b) To gain perspectives and insights beyond those acquired during data charting and analysis, as well as suggestions for knowledge translation.
(c) To present the preliminary findings of the scoping review to research and policy stakeholders in the field.

The insights acquired throughout this phase will be analysed, interpreted and integrated into the review findings and recommendations.

### Study status

At the time of publication of this protocol, informal preliminary searches of the literature had been undertaken primarily to help to identity keywords, phrases and index terms in order to develop the full search strategy.

## Conclusion and dissemination

The overarching aim of the scoping review is to gain a comprehensive understanding of the literature on risk factors for suicide among PWUD over the past two decades. A scoping review will achieve several important outcomes that will contribute to the overall progress and direction of this field, including a narrative overview of the types of evidence available, a thematic summary of what is currently known about risk factors for suicide among PWUD, identification of knowledge gaps and research limitations, and recommendations for future research avenues, policy development and professional practice. To our knowledge, this will be the first scoping review of risk factors for suicide among this particularly high-risk group. Key strengths of the review will be the use of the most up-to-date methodological guidelines and recommendations ^37, 39, 40, 47– 49^ and the process of stakeholder consultation. A limitation of the scoping review methodology is that it does not typically include an assessment of publication bias or research quality appraisal ^39^; however, relative to other types of reviews such as a systematic review, a scoping review has the capacity to capture the breadth of available evidence on a given topic from a large variety of sources including grey and unpublished literature, ascertain the types of evidence available across the body of literature and make appropriate recommendations for future research, policy and practice ^50^.

The results of the scoping review will be disseminated widely. The review will be submitted for publication in a peer reviewed academic journal, presented at an interdisciplinary conference and disseminated to key experts and stakeholders, including those identified during the review process as well as those in the network of the review team. We also plan to submit a blog post to a major non-profit organisation such as *Addaction* to disseminate to the wider non-academic audience.

## Data availability

No data are associated with this article.
