# Supplementary material for: Risk factors for completed suicide among people who use drugs: A scoping review protocol
Source: HRB Open Res. 2021 May 17;3:45. Originally published 2020 Jul 17. [Version 3] doi: 10.12688/hrbopenres.13098.3 (PMC7885289; doi:10.12688/hrbopenres.13098.3)
Supplement: Peer review history

# Peer review: Reviewer response for version 3

**DOI:** 10.21956/hrbopenres.14400.r29468

**Published:** 2021-06-02

**Authors:** Alexandra Pitman

**Affiliations:** 1 Division of Psychiatry, University College London, London, UK; 2 Camden and Islington NHS Foundation Trust, London, UK

I have reviewed the amendments and the responses seem fine apart from one issue. Although the authors have justified not conducting quality appraisal of included articles, as this is a scoping review, they justify this alongside omitting an assessment of publication bias, which is a very different process. As a scoping review is a perfect opportunity to assess publication bias it would be important to include this too.

Is the study design appropriate for the research question?

Yes

Is the rationale for, and objectives of, the study clearly described?

Yes

Are sufficient details of the methods provided to allow replication by others?

Yes

Are the datasets clearly presented in a useable and accessible format?

Not applicable

Reviewer Expertise:

Suicide and self-harm epidemiology.

I confirm that I have read this submission and believe that I have an appropriate level of expertise to confirm that it is of an acceptable scientific standard.

# Peer review: Reviewer response for version 2

**DOI:** 10.21956/hrbopenres.14325.r28779

**Published:** 2021-02-15

**Authors:** Alexandra Pitman

**Affiliations:** 1 Division of Psychiatry, University College London, London, UK; 2 Camden and Islington NHS Foundation Trust, London, UK

This study protocol describes the plan to conduct a scoping review of the literature on risk factors for suicide among people who use drugs. It has already had a first round of peer reviewer comments, to which the authors have responded appropriately. The key compromise, as with all scoping reviews, lies in covering the breadth of research but at the expense of time available to conduct critical appraisal of included articles. This has been commented on by other reviewers and responded to.

 The searches will start from a date set at 2000, yet as other reviewers point out this seems quite late, and would miss out potentially useful studies. The rationale for this date is that in a previous similar review most of the studies identified were published after 2000, and that the author of a EMCDDA report had observed differences in the patterns of drug use since 1995. However this does not seem a robust justification, unless people had stopped using certain substances. I would suggest an earlier search limit of 1980 given that the outcome is restricted to completed suicide.

 A couple of minor comments:

 Page 6 mentions thematic analysis as a way of summarising the literature, but this is a qualitative method and would be more appropriate as a narrative synthesis or summary.   

 Dissemination plans would be improved if a blog was planned with a major non-profit (e.g. Addaction) to gain wide reach beyond academic audiences.

Is the study design appropriate for the research question?

Yes

Is the rationale for, and objectives of, the study clearly described?

Yes

Are sufficient details of the methods provided to allow replication by others?

Yes

Are the datasets clearly presented in a useable and accessible format?

Not applicable

Reviewer Expertise:

Suicide and self-harm epidemiology.

I confirm that I have read this submission and believe that I have an appropriate level of expertise to confirm that it is of an acceptable scientific standard, however I have significant reservations, as outlined above.

# Response

**Published:** 2021-02-16

**Authors:** Lisa Murphy

**Affiliations:** Health Research Board, Ireland

**This study protocol describes the plan to conduct a scoping review of the literature on risk factors for suicide among people who use drugs. It has already had a first round of peer reviewer comments, to which the authors have responded appropriately. The key compromise, as with all scoping reviews, lies in covering the breadth of research but at the expense of time available to conduct critical appraisal of included articles. This has been commented on by other reviewers and responded to.**

 We thank the reviewer for their time and suggestions, which we believe have improved the quality of the manuscript and proposed review.

**The searches will start from a date set at 2000, yet as other reviewers point out this seems quite late and would miss out potentially useful studies. The rationale for this date is that in a previous similar review most of the studies identified were published after 2000, and that the author of a EMCDDA report had observed differences in the patterns of drug use since 1995. However, this does not seem a robust justification, unless people had stopped using certain substances. I would suggest an earlier search limit of 1980 given that the outcome is restricted to completed suicide.**

 We appreciate the reviewer’s point regarding the timeframe. Our rationale for restricting the timeframe is based on several points worth noting:

1. There have been notable changes in drug use patterns and trends over time. Of particular note is a systematic review of literature on the topic of non-medical use of prescription drugs; although no date limitations were applied, the review found that most of the literature was post 2000 (Clark et al., 2015*). Moreover, a recent article from the EMCDDA* showing a change in drug use patterns over time states: ‘The world is a very different place in 2020 than it was in 1995’, ‘Strikingly, across the 25 years of reporting, cannabis has gone from being a drug relatively rarely reported in data from drug treatment registries to being the drug that is now most commonly associated with new treatment entries’. We believe that such changes in drug use patterns, as well as changes across almost all other aspects of society over time, including policy, practice and health-related services, growing socio-economic inequality, etc. are relevant to the study of suicide as they hold implications for how we characterise and assess risk among different populations, including people who use drugs. The findings of this study should support evidence-based knowledge to influence policy and practise that is relevant to help prevent deaths due to suicide among people who currently use drugs.
2. Particularly pertinent studies that remain relevant today (i.e. seminal papers published before the year 2000) that continue to be cited in the literature will be identified in the full text searching and data extraction stages and considered for inclusion in the review on a case by case basis according to our search and inclusion criteria. This has now been noted in the manuscript (in the ‘Eligibility criteria’ subsection).
3. While we have limited our inclusion criteria to publications from 2000 onwards, many studies published in the early part of the century (2000 to 2005) will include data from preceding years therefore we are confident any pertinent information will be captured within the timeframe of publications in the last twenty years. From our initial searches, many of the published studies we have identified describe data collected in the period immediately preceding 2000. These data are typically included in large scale studies published in 2000 or thereafter and will be included if relevant.

*Clark, M., Hamdi-Ghoz, E., Jauffret-Roustide, M., Le Moigne, P., Melpomeni Malliori, M., Simeoni, E., Jasaitis, E., Jovanovic, M., James, K. & Palczak, K. 2015a. The gender dimension of non-medical use of prescription drugs in Europe and the Mediterranean region. *Pompidou Group and the Council of Europe.* Strasbourg: Council of Europe, Co-operation Group to Combat Drug Abuse and Illicit Trafficking in Drugs (Pompidou Group).

 *Looking back on 25 years of annual reporting on the drugs problem in Europe By Paul Griffiths, EMCDDA Scientific Director 

**A couple of minor comments:**

**Page 6 mentions thematic analysis as a way of summarising the literature, but this is a qualitative method and would be more appropriate as a narrative synthesis or summary.**

 Thank you for flagging this, it has now been re-worded (‘Stage 5’ subsection, lines 4 and 9).

**Dissemination plans would be improved if a blog was planned with a major non-profit (e.g. Addaction) to gain wide reach beyond academic audiences.**

 We agree with the reviewer and have now included submitting a blog post as part of the dissemination plan (final paragraph of the manuscript).

# Peer review: Reviewer response for version 2

**DOI:** 10.21956/hrbopenres.14325.r28526

**Published:** 2021-02-08

**Authors:** Anne Reneflot

**Affiliations:** 1 Mental and Physical Health, Norwegian Institute of Public Health, Skøyen, Oslo, Norway

I have no further comments.

Is the study design appropriate for the research question?

Yes

Is the rationale for, and objectives of, the study clearly described?

Partly

Are sufficient details of the methods provided to allow replication by others?

Yes

Are the datasets clearly presented in a useable and accessible format?

Not applicable

Reviewer Expertise:

Suicide, epidemiology, demography.

I confirm that I have read this submission and believe that I have an appropriate level of expertise to confirm that it is of an acceptable scientific standard.

# Peer review: Reviewer response for version 1

**DOI:** 10.21956/hrbopenres.14200.r28003

**Published:** 2020-11-03

**Authors:** Anne Reneflot, Kim Stene-Larsen

**Affiliations:** 1 Mental and Physical Health, Norwegian Institute of Public Health, Skøyen, Oslo, Norway

Thank you for the opportunity to review the paper *Risk factor for completed suicide among people who use drugs: A scoping review protocol*

 This paper presents a protocol for a scoping review of the risk factors for completed suicide among people who use drugs. The increased suicide risk associated with problematic drug use is well documented, and the planned scoping review has the potential to make an important contribution to the existing literature. However, we believe the protocol would benefit from some important revisions:

1. **Objectives of the review**: The third objective of the planned scoping review is *to inform suicide prevention policy and best practice guidelines for working with PWUD. *However, this objective is considerably limited by the lack of quality assessment of eligible studies included in the planned scoping review. We find it particularly problematic that the authors plan to include unpublished and grey literature. This challenge should be acknowledged, and the authors must either adjust the third objective of the planned scoping review, ensure a kind of quality appraisal of the included studies or conduct a systematic review instead.
2. **Definition of the population**. We find the protocol unclear as to which population this scoping review will cover. Will the planned scoping review focus on people who use drugs or on people with problematic drug use? Will the former definition of the population also  include people with recreational drug use?  We believe the planned scoping review would benefit from limiting the population to people with problematic drug use or a drug abuse disorder. With a very broadly defined population, it will be challenging to identify risk factors particularly pertaining to groups with a high risk of suicide. Further, we question why the research group have decided to exclude people with problematic alcohol use and alcohol use disorder? Is it reasons to believe that the risk factors for suicide in this group is very different from those with a problematic drug use and drug use disorders? At least, we believe this should be discussed in more detail in the protocol.
3. **Eligibility criteria**: The authors have decided to limit the search from 2000 and onwards. Is it reason to believe that risk factors for completed suicide among people who use drugs have changed after the turn of the century? By excluding studies published before year 2000 the authors risk to miss seminal studies. Further, evidence sources with obvious overlap in either participant samples or datasets, only sources that provide the most information relevant to the aims of the scoping review will be prioritized. This is common in systematic reviews, but in a scoping review the aim is among others to map the existing literature and we believe this restriction is unnecessary.
4. **Outcome**: in the planned scoping review, only studies that examine risk factors associated with completed suicide will be included. A problem with this strategy is that the authors risk ending up with very few included studies. We acknowledge that the risk factors for completed suicide and non-fatal suicidal behavior may differ, but suicide attempt is an important risk factor and precursor for suicide and it may therefore be informative to include studies of risk factors associated with suicide attempts as well.

Is the study design appropriate for the research question?

Yes

Is the rationale for, and objectives of, the study clearly described?

Partly

Are sufficient details of the methods provided to allow replication by others?

Yes

Are the datasets clearly presented in a useable and accessible format?

Not applicable

Reviewer Expertise:

Suicide, epidemiology, demography.

We confirm that we have read this submission and believe that we have an appropriate level of expertise to confirm that it is of an acceptable scientific standard, however we have significant reservations, as outlined above.

# Response

**Published:** 2020-11-20

**Authors:** Lisa Murphy

**Affiliations:** Health Research Board, Ireland

We would like to thank both reviewers for their careful read and review of our manuscript. We know that this takes significant time and effort and appreciate the recommendations they have made, which we believe have improved the overall quality of the protocol.

**Reviewer 1: Holly Wilcox**

**1. In the Background section of the Abstract suicide add "AMONG" or "in" PWUD.**

 Many thanks for catching this error, we have now included ‘among’.

**2. The intro reports old data - most recent data is from 2018 not 2016.**

 Assuming the reviewer is referring to WHO data, we have checked the most recent reports and information from the WHO regarding suicide and believe that the 2016 report is the most recent to specifically highlight suicide as accounting for 1.4% of deaths worldwide and being the 18 ^th^ leadings cause of death globally. Subsequent reports that state the same cite this 2016 report. The most recent information provided in a 2019 WHO factsheet indicates that 800,000 deaths can be attributed to suicide and is cited at the very beginning of the introduction section.

**3. Great that you are including grey literature and including stakeholders. Who are the stakeholders? Do they include people with lived experience of drug use and suicide attempts or family member who lost a loved one to suicide and drug overdose?**

 The stakeholders are identified in the ‘Stage 6’ subsection:

 “ *Consultations will occur with national experts from the Irish National Drug-Related Deaths Index (NDRDI) Steering Committee…the Technical Advisory Group of the National Office of Suicide Prevention and international experts from the European Monitoring Centre for Drugs and Drug Addiction (EMCDDA) and the World Health Organization.*” 

 While we very much appreciate the importance of exploring the lived experience of suicide attempts among people who use drugs, it is not the focus of this review. We hope that this review can serve as a steppingstone to continued work on this topic, including exploration of risk factors for attempted suicide among people who use drugs.

 Regarding consultation with family members, we have now made clearer in the protocol where this form of consultation will occur (in the ‘Stage 6’ subsection):

 “ *Consultations will occur with national experts from the Irish National Drug-Related Deaths Index (NDRDI) Steering Committee, which includes community representation for families affected by drug-related deaths (including suicides)*…”

**4. Age range of the population could differ by risk factors.**

 We agree with the reviewer and have now noted this in the protocol as a factor to consider when mapping and analysing the data (paragraph 2 in the ‘Stage 5’ subsection).

**5. In terms of the population will you include those with alcohol use/misuse/abuse? Will you include those who try to intentionally overdose to kill themselves?**

 The purpose of the review is to examine risk factors for complete suicide among PWUD, which includes people who use both drugs and alcohol, but not people who use/misuse/abuse alcohol only. This is noted in Table 1 which outlines inclusion exclusion criteria (under ‘Population’).

 Regarding suicide methods, including sources that refer to intentional overdose, this is also detailed in Table 1 (under ‘Outcome’).

**6. It would be ideal to separately list modifiable and non-modifiable risk factors as modifiable risk factors are actionable; it would be ideal to also draw a distinction between proximal and distal risk factors.**

 Many thanks for this suggestion. We have included a note on (paragraph 2 in the ‘Stage 5’ subsection) to indicate that we intend to characterise risk factors according to these categories during the data charting and analysis process.

**7. Will this be done for each drug or drug users lumped together? Impact of the review would be better if results were provided by drug and multidrug users.**

 Thank you for this suggestion. We agree and have now included a note that when possible, trends will be analysed according to specific drugs as well as the number of drugs reported (paragraph 2 in the ‘Stage 5’ subsection). However, we note that this depends on the level of detail included in the studies included in the review.

**8. These groups are ok but if you want this to have international impact (not just Ireland or Europe), WHO seems better: Consultations will occur with national experts from the Irish National Drug-Related Deaths Index (NDRDI) Steering Committee, the Technical Advisory Group of the National Office of Suicide Prevention and international experts from the European Monitoring Centre for Drugs and Drug Addiction (EMCDDA). Those with lived experience should provide input.**

 Many thanks to the reviewer for drawing our attention to the fact that this stage of the review process is not described clearly in the protocol. We are contacting experts and organisations/groups worldwide, including people at the WHO. This is now revised on in the ‘Stage 6’ subsection.

 Regarding including those with lived experience, we refer to our response to the reviewers’ point no. 3 above.

**9. It would be ideal to include an assessment of publication bias or research quality appraisal.**

 Thank you for raising this point. One of the principals of a scoping review is not to include a quality appraisal of the items included, although we acknowledge that this is debated in the literature. We also acknowledge that some researchers conducting scoping reviews are incorporating quality appraisal into their protocol. However, we believe this should be determined on a case by case basis. For our purposes, it is important to obtain a broad sense of the field, given that there has been little empirical research to date. In this sense, we anticipate that a substantial proportion of our final sources will come from the grey literature - including policy papers, reports, expert input etc. We believe that quality appraisal would not make a substantial contribution to the overall findings of this particular review. However, we will acknowledge this in the discussion section of the review manuscript.

**Reviewer 2 & 3: Anne Reneflot & Kim Stene-Larsen**

**1. Objectives of the review: The third objective of the planned scoping review is to inform suicide prevention policy and best practice guidelines for working with PWUD. However, this objective is considerably limited by the lack of quality assessment of eligible studies included in the planned scoping review. We find it particularly problematic that the authors plan to include unpublished and grey literature. This challenge should be acknowledged, and the authors must either adjust the third objective of the planned scoping review, ensure a kind of quality appraisal of the included studies or conduct a systematic review instead.**

 Many thanks for raising this point. One of the principals of a scoping review is not to include a quality appraisal of the items included, although we acknowledge that this is debated in the literature. We also acknowledge that some researchers conducting scoping reviews are incorporating quality appraisal into their protocol, but we believe this should be determined on a case by case basis. We have, however, revised the phrasing of the third objective in the Introduction.

 For our purposes, it is important to obtain a broad sense of the field given that there has been little empirical / published research to date (hence, limited knowledge can be garnered from the published literature alone). In this sense, we anticipate that a substantial proportion of our final sources will come from the grey literature - including policy papers, government reports, expert input etc, which are used to inform policy in this area, including prevention policy. This is acknowledged in the ‘Conclusion and Dissemination’ section at the end of the protocol and will also be acknowledged in the discussion section of the review manuscript.

**2. Definition of the population. We find the protocol unclear as to which population this scoping review will cover. Will the planned scoping review focus on people who use drugs or on people with problematic drug use? Will the former definition of the population also include people with recreational drug use?  We believe the planned scoping review would benefit from limiting the population to people with problematic drug use or a drug abuse disorder. With a very broadly defined population, it will be challenging to identify risk factors particularly pertaining to groups with a high risk of suicide. Further, we question why the research group have decided to exclude people with problematic alcohol use and alcohol use disorder? Is it reasons to believe that the risk factors for suicide in this group is very different from those with a problematic drug use and drug use disorders? At least, we believe this should be discussed in more detail in the protocol.**

 Many thanks to the reviewers for raising this point. While we agree that there is a lack of clarity regarding the population, the inconsistent use of terms in the literature related to people who used drugs makes it quite difficult (particularly in the first stages of the review process) to develop a strict inclusion/exclusion criterion around population type. For this reason, we have kept the parameters of the population deliberately broad. Once we have identified suitable sources, we will be in a better position to categorise and analyse findings according to population type, e.g. studies that specifically focus on people with drug dependence or drug/substance use disorder compared to those that refer to the population sample (or a subgroup of the sample) as ‘drug users’ or ‘people who use drugs’, without reference to drug use severity. We have now included a note on this in paragraph 2 in the ‘Stage 5’ subsection.

**3. Eligibility criteria: The authors have decided to limit the search from 2000 and onwards. Is it reason to believe that risk factors for completed suicide among people who use drugs have changed after the turn of the century? By excluding studies published before year 2000 the authors risk to miss seminal studies. Further, evidence sources with obvious overlap in either participant samples or datasets, only sources that provide the most information relevant to the aims of the scoping review will be prioritized. This is common in systematic reviews, but in a scoping review the aim is among others to map the existing literature and we believe this restriction is unnecessary.**

 While we appreciate the reviewer’s point regarding the timeframe, our rationale for restricting the timeframe is based on notable changes in drug use patterns and trends over time. Of particular note is a systematic review of literature on the topic of non-medical use of prescription drugs; although no date limitations were applied, the review found that most of the literature was post 2000 (Clark et al., 2015*). Moreover, a recent article from the EMCDDA* showing a change in drug use patterns over time states: ‘The world is a very different place in 2020 than it was in 1995’  ‘Strikingly, across the 25 years of reporting, cannabis has gone from being a drug relatively rarely reported in data from drug treatment registries to being the drug that is now most commonly associated with new treatment entries’.

 We believe that such changes in drug use patterns, as well as changes across almost all other aspects of society over time, including policy, practice and health-related services, growing socio-economic inequality, etc. are relevant to the study of suicide as they hold implications for how we characterise and assess risk among different populations, including people who use drugs.

 *Clark, M., Hamdi-Ghoz, E., Jauffret-Roustide, M., Le Moigne, P., Melpomeni Malliori, M., Simeoni, E., Jasaitis, E., Jovanovic, M., James, K. & Palczak, K. 2015a. The gender dimension of non-medical use of prescription drugs in Europe and the Mediterranean region. *Pompidou Group and the Council of Europe.* Strasbourg: Council of Europe, Co-operation Group to Combat Drug Abuse and Illicit Trafficking in Drugs (Pompidou Group).

 *Looking back on 25 years of annual reporting on the drugs problem in Europe By Paul Griffiths, EMCDDA Scientific Director.  

 In regards excluding evidence sources with obvious overlap, we emphasise on page 7 (under ‘eligibility criteria’) that any evidence sources that do not provide new information will be excluded. We will retain any sources that provide new insights, even if they use the same data. In instances where two sources draw from the same data and one does not provide any additional insight to the other, we will exclude it. We do not anticipate this to be a major obstacle, however, given the limited amount of empirical work that appears to have been done to date.

**4. Outcome: in the planned scoping review, only studies that examine risk factors associated with completed suicide will be included. A problem with this strategy is that the authors risk ending up with very few included studies. We acknowledge that the risk factors for completed suicide and non-fatal suicidal behaviour may differ, but suicide attempt is an important risk factor and precursor for suicide and it may therefore be informative to include studies of risk factors associated with suicide attempts as well.**

 Many thanks to the reviewers for this suggestion. We agree that ultimately, we may end up with few empirical studies / evidence sources. However, we believe that limiting the review to studies of completed suicide provides a clear purpose and scope. Mapping the available literature regarding risk factors for attempted suicide among PWUD is a very worthwhile but ultimately distinct research endeavour, particularly, as the reviewer notes, since the nature and trends of risk factors will vary depending on the outcome of interest. We hope that this review can serve as a steppingstone to continued work on this topic, including exploration of risk factors for attempted suicide among people who use drugs.

# Peer review: Reviewer response for version 1

**DOI:** 10.21956/hrbopenres.14200.r27897

**Published:** 2020-10-26

**Authors:** Holly Wilcox

**Affiliations:** 1 Department of Mental Health, Johns Hopkins Bloomberg School of Public Health, Baltimore, MD, USA

In the Background section of the Abstract suicide add "AMONG" or "in" PWUD.

 The intro reports old data - most recent data is from 2018 not 2016.

 Great that you are including grey literature and including stakeholders. Who are the stakeholders? Do they include people with lived experience of drug use and suicide attempts or family member who lost a loved one to suicide and drug overdose?

 Age range of the population could differ by risk factors.

 In terms of the population will you include those with alcohol use/misuse/abuse? Will you include those who try to intentionally overdose to kill themselves?

 It would be ideal to separately list modifiable and non modifiable risk factors as modifiable risk factors are actionable; it would be ideal to also draw a distinction between proximal and distal risk factors.

 Will this be done for each drug or drug users lumped together? Impact of the review would be better if results were provided by drug and multidrug users.

 These groups are ok but if you want this to have international impact (not just Ireland or Europe), WHO seems better: Consultations will occur with national experts from the Irish National Drug-Related Deaths Index (NDRDI) Steering Committee, the Technical Advisory Group of the National Office of Suicide Prevention and international experts from the European Monitoring Centre for Drugs and Drug Addiction (EMCDDA). Those with lived experience should provide input.

 It would be ideal to include an assessment of publication bias or research quality appraisal.

Is the study design appropriate for the research question?

Partly

Is the rationale for, and objectives of, the study clearly described?

Partly

Are sufficient details of the methods provided to allow replication by others?

Partly

Are the datasets clearly presented in a useable and accessible format?

Not applicable

Reviewer Expertise:

Suicide prevention.

I confirm that I have read this submission and believe that I have an appropriate level of expertise to confirm that it is of an acceptable scientific standard, however I have significant reservations, as outlined above.
